# Digital teaching tools and global learning communities

**DOI:** 10.12688/f1000research.6150.2

**Published:** 2015-07-20

**Authors:** Mary Williams, Patti Lockhart, Cathie Martin

**Affiliations:** 1 American Society of Plant Biologists, Rockville, MD, 20855, USA; 2 John Innes Centre, Norwich Research Park, Norwich, NR4 7UH, UK

**Keywords:** Plant biology, science, education online, education digital, education global, education

## Abstract

In 2009, we started a project to support the teaching and learning of university-level plant sciences, called Teaching Tools in Plant Biology. Articles in this series are published by the plant science journal, *The Plant Cell* (published by the American Society of Plant Biologists). Five years on, we investigated how the published materials are being used through an analysis of the Google Analytics pageviews distribution and through a user survey. Our results suggest that this project has had a broad, global impact in supporting higher education, and also that the materials are used differently by individuals in terms of their role (instructor, independent learner, student) and geographical location. We also report on our ongoing efforts to develop a global learning community that encourages discussion and resource sharing.

## Introduction

More than 20 years ago, the first graphical web browser *Netscape Navigator* was released, and the world changed forever ( Mosaic Communications press release, 1994). Widespread access to the repository of information stored on computers across the globe has changed the way we teach, learn, and communicate. The Internet opened the door to global educational tools, from Wikipedia to massively open online courses (aka MOOCs), and changed the way that students access and engage with information. The opportunities afforded by this international connectedness are still being developed and explored. We describe an innovative, ongoing project to create and disseminate university-level educational materials in plant science by way of their publication in a scholarly journal, we report the new ways these resources are being used across the globe, and we propose new opportunities for enhanced engagement.

## Why is plant science important? Food production is a global challenge

We face many challenges in the 21 ^st^ century as a consequence of rising population, rising affluence, and energy requirements and global climate changes. It is widely acknowledged that food production must increase by 50% or more by 2050 ( Ray *et al.*, 2013), and at the same time it is vital to preserve natural ecosystems and employ more sustainable food production methods. Food production is a global problem that must be addressed locally, and plant scientists, horticulturalists, and agronomists with local knowledge will be at the forefront of these efforts.

As a first step to addressing the need for increased recruitment and training of plant scientists, the premier plant science journal, *The Plant Cell* (published by the American Society of Plant Biologists) in 2009 began publishing online educational tools for use in higher education. These materials are presented as a series of articles called “ Teaching Tools in Plant Biology”. To date we have published 30 Teaching Tools; each includes a set of image-rich PowerPoint slides from which educators can select parts or complete teaching materials, a review-style article written for advanced undergraduates, recommended reading lists that span recent research and review articles as well as historical reports, and teaching guides with questions for discussion and assessment. Each Teaching Tool is peer-reviewed and regularly updated with content and references, features that were impossible before online publishing.

The overarching goal of this project is to support plant scientists in their teaching and learning. The targeted users of these resources are the readers of *The Plant Cell* (access to the articles requires a personal or institutional subscription to the journal) and their students in upper-level undergraduate or graduate courses. Besides summarizing current plant science, specific objectives are to support a variety of teaching and learning styles, to highlight the relevance of plant research to global issues, and to help bridge the gap between textbook-based learning and learning from the primary literature that occurs as students transition from undergraduate to graduate education.

**Figure 1.**
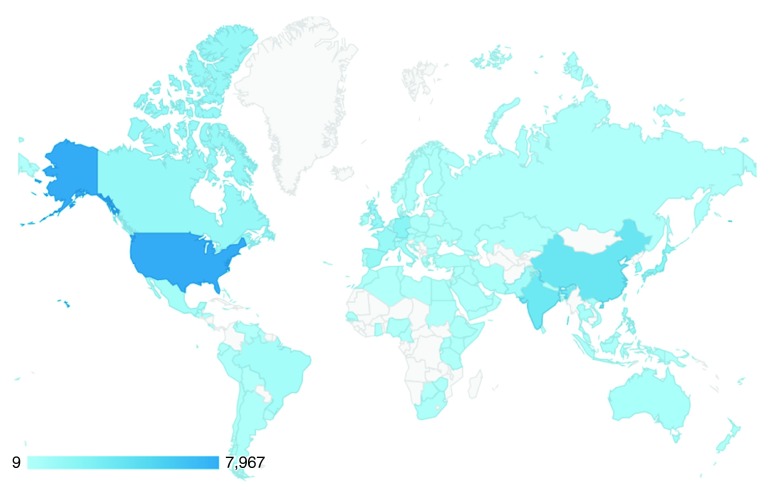
Geographic distribution of Teaching Tools pageviews during 2014, provided by Google Analytics. More than 100 countries are represented in the more than 250,000 pageviews accrued during 2014. Since 2011, the US, China and India have been the countries with the largest number of pageviews.

As online resources, these are used widely across the globe and have been regularly accessed from more than 100 countries. In 2014, the collection received more than 250,000 pageviews. The countries with the greatest number of pageviews were the United States, China, India, Germany, Japan, S. Korea, the United Kingdom, Spain, Canada, and France ( Figure 1); each of these countries falls within the top 20 countries in terms of number of Internet users ( Internet World Stats, 2014). Although we can track download numbers and sources, we lacked data about *who* is reading these articles and *how* they are being used. Therefore, four years into the project, we conducted a survey to learn more about how these resources are used in different countries by instructors, students, and independent learners. A link to the survey was embedded on the download page for the Teaching Tools resources. To encourage survey completion, we provided a $100 Amazon voucher to one randomly selected survey participant.

We received nearly 300 completed surveys representing input from 50 countries. About half of our responders were instructors in a course. Two-thirds of these were from ten countries, in order: United States (28), India (17), Spain (12), China (10), Mexico (9), France (7), Brazil (7), UK (7), Argentina (6), Germany (5), with four or fewer respondants from 32 additional countries. The other half of the respondants were students in a formal course (57 total, with 15 from China, 7 from India, 5 from Mexico, 4 from United States, and fewer from 16 other countries) or independent learners not enrolled in a formal course (88 total, with 23 from China, 19 from USA, 12 from India and fewer from 20 additional countries). Thus, the survey respondants reasonably represented the users of Teaching Tools in Plant Biology as determined by pageviews.

## Teaching Tools in Plant Biology fills a gap in plant science educational resources

Many online resources are available for students and teachers of biology. However, because plant science remains a relatively small part of most primary, secondary and introductory-level university curricula, and plant science garners less funding support than many other biology disciplines, the number of educational resources for plant biology remains small compared to other topics. As an illustration, the Life Science Teaching Resource Community currently links to 24 plant biology lessons, 8 soil biology lessons, 188 invertebrate biology lessons, 231 vertebrate biology lessons, 655 neurobiology lessons, and 6215 animal physiology lessons. Plant sciences can easily get lost or overlooked in general biology sites such as this and others.

The American Society of Plant Biologists has been a long-time supporter of and partner in the development of learning materials for plant biology, and maintains a curated list of links to plant science educational resources ( K-12 resources). An early ASPB supported project was the “ 12 Principles for Plant Biology”, a set of concepts for primary- and secondary-level plant science education. More recently, ASPB in partnership with the Botanical Society of America (BSA) developed a set of “ Core Concepts and Learning Objectives in Plant Biology for Undergraduates”, which tie in with the recommendations of the National Science Foundation Vision and Change and the framework for primary and secondary education developed by the National Academy of Sciences Board on Science Education ( Quinn *et al.*, 2012).

Ample evidence points to the importance of inquiry and investigation in learning. Plants are well-suited for classroom based hands-on activities, and several excellent programs support teachers and learners. Examples include Wisconsin Fast Plants, which distributes resources, materials and seeds to support hands-on activities, ABRC (Arabidopsis Biological Resource Center) Education and Outreach portal, which distributes protocols and seeds of the model plant *Arabidopsis thaliana* for inquiry-based activities, Science and Plants for Schools , which distributes resources for activities and practicals as well as videos, career advice and more ( Jenkins, 2015), and 12 Activities for 12 Principles of Plant Biology, guides for hands-on inquiries for exploring the concepts developed as the 12 Principles of Plant Biology. PlantingScience.org is a program developed by the BSA with the support of ASPB which partners primary and secondary student research teams with scientist mentors. This program benefits the students who have the opportunity to interact with a scientist and glean their expertise in the design and interpretation of their experiment, but also the mentors who have an opportunity for hands-on experience in science education ( Adams & Hemingway, 2014; Hemingway *et al.*, 2015). Additional plant science resources have been developed by The Gatsby Charitable Foundation in the form of The Plant Science Tree ( http://www.tree.leeds.ac.uk/), which provides educational resources in the form of online lectures, lecture slides, movies, practicals and images contributed from the research community ( Levesley *et al.*, 2012; Levesley *et al.*, 2014), and an inquiry-based laboratory module has been published as “The Plant Detective’s Manual”, developed by scientists at the Australian National University ( Estavillo *et al.*, 2014). 

Although these core concepts and inquiry-based activities are based on good pedagogical principles and can inspire students to want to learn more about plant science, they rarely prepare students for the rigor and complexity of contemporary plant science research. There is a huge knowledge gap between “ Plants are the primary food and oxygen producers on Earth” and “ Flavodiiron protein Flv2/Flv4-related photoprotective mechanism dissipates excitation pressure of PSII in cooperation with phycobilisomes in cyanobacteria”, and it is this gap between engagement and expertise that Teaching Tools in Plant Biology is designed to bridge, by making the primary literature accessible.

## Teaching Tools are used in many different ways

**Figure 2.**
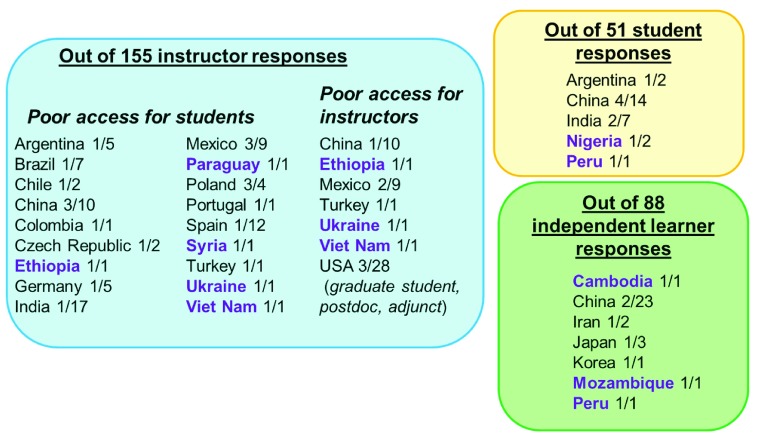
Between 6–20% of respondents in each category reported poor access to textbooks for themselves or their students. This was particularly a concern for respondents from least-developed countries (shown in purple) from which access Teaching Tools in Plant Biology is available through AGORA (Access to Global Online Research in Agriculture).

When we initiated this series, we envisioned the articles being used as a supplement to a textbook, and intentionally did not tie them to the content of any of the several textbooks used to teach advanced plant biology or plant physiology. Through our survey, we learned that in some cases both instructors and students have poor access to textbooks (due to financial or language issues) and many are using the materials from *The Plant Cell* as a substitute for a textbook ( Figure 2). A particularly high proportion of survey respondents from least-developed countries who access the materials through Research4Life/AGORA identify as having poor access to textbooks. Now that we recognize that these articles are in some cases being used in lieu of a textbook, we are including more basic as well as advanced coverage of each topic.

**Figure 3.**
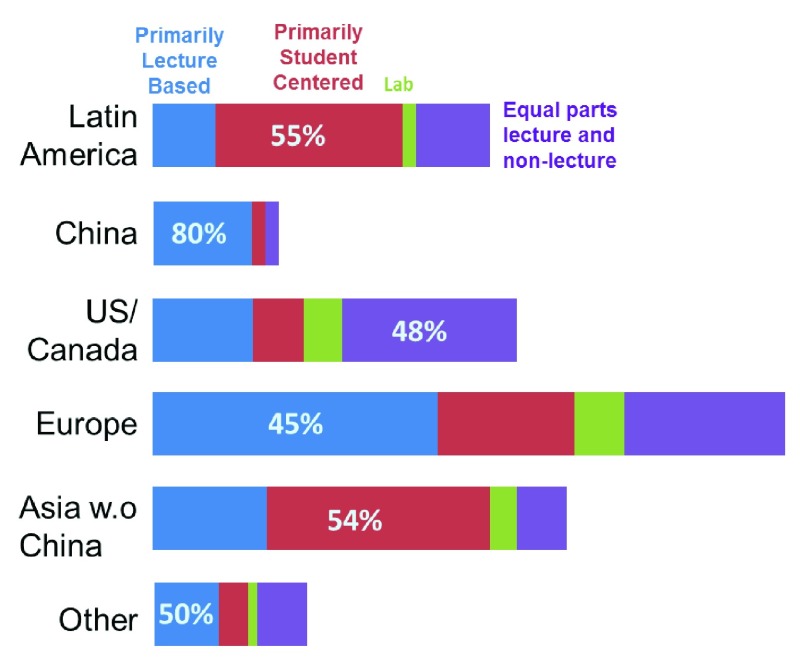
The type of course in which Teaching Tools in Plant Biology materials are used varies for many reasons and depends on institutional, political and cultural practices as well as instructor preferences. This question was only asked to instructors, who selected one category out of the four options indicated.

Our survey also reinforced the fact that teaching styles and approaches are quite varied. Although in the US there is wide support from learned societies, funding agencies, and many instructors for an emphasis on student-centered, project-based learning, this approach is not universally embraced, particularly outside the US ( AAAS, 2011; Freeman *et al.*, 2014; Tanner, 2009). For example, in China higher education styles tend to be more traditional for a variety of social, cultural, and political reasons ( Lee, 2004; Thompson & Ku, 2005; Zhang, 2007; Zhang, 2010). In line with this, 80% of the survey respondents in China reported that the materials are used in courses that are primarily lecture based, whereas in Asia (without China) and Latin America, the largest category of course types was described as “primarily student centered” (54% and 55%) ( Figure 3). Respondents from the US or Canada mainly identified their courses “equal parts lecture and non-lecture”.

**Figure 4.**
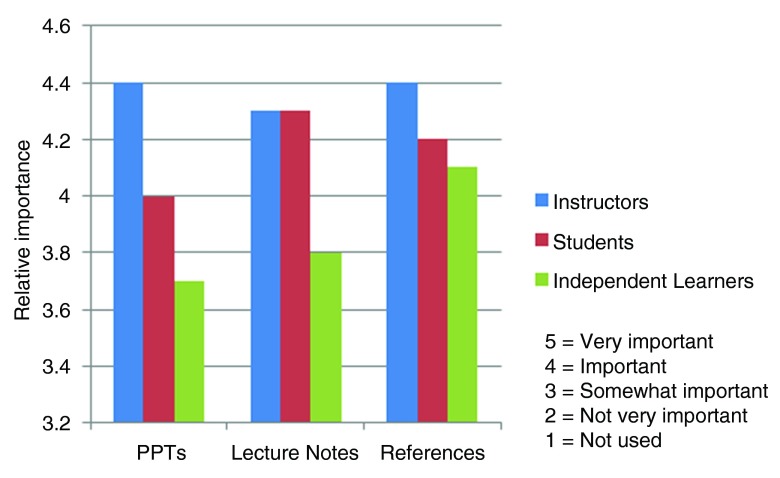
Instructors, students and independent learners have different priorities for the importance of the PowerPoint files, Lecture Notes and References provided in the Teaching Tools. Instructors particularly value the PowerPoint files as sources of images for teaching, the students rank the Lecture Notes as most important, and the independent learners rank the references as most important.

Our survey responses also indicated that the Teaching Tools are being used differently by students, independent learners, and instructors ( Figure 4). Course instructors most highly valued the PowerPoint files as sources of images for preparing lectures, students found the Lecture Notes important, and independent learners rated the references as the most important component (independent learner respondents included undergraduates, graduate students, postdocs, lab heads, staff scientists, teaching staff, and other, a category that included librarian, communicator, and entrepreneur).

Although English is the lingua franca of science, about half of the people we surveyed teach or learn in a different language, raising the question of whether we should present these materials in multiple languages. As a first step, we have coordinated the production of translations of one of these lessons, called “Why Study Plants?” into 14 languages ( http://www.plantcell.org/site/teachingtools/TTPB1.xhtml), and we are exploring the possibility of translating additional lessons in collaboration with the Global Plant Council.

## Harnessing social media to develop a global learning community

**Figure 5.**
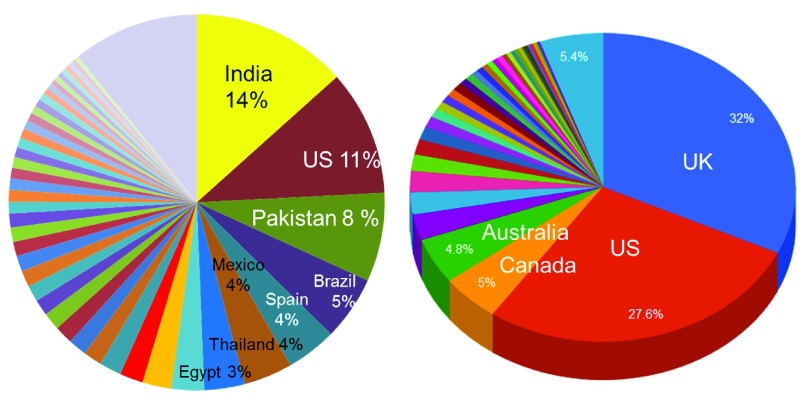
As of December 2014, the geographical origins of followers of Teaching Tools in Plant Biology feeds on Facebook (left, >3600 followers) and Twitter (right, >2200 followers) are quite different, with Twitter followers showing a strong bias towards countries whose primary language is English.

We have been exploring the use of social media as a way to connect the geographically widespread users of Teaching Tools in Plant Biology. Scientists are generally considered slow adopters of social media, but there is growing awareness that these tools can be used in a professional context and support professional development ( Bik & Goldstein, 2013; Osterrieder, 2013). Since the earliest days of online education, educators have stressed the importance of building community and trust ( Brown, 2001; Rovai, 2002); trust is based on positive interactions, whether online or in person. Social media provide the opportunities for community building that were lacking in the earliest iterations of distance learning and have proven to be an effective medium to support interactions among globally distributed professional communities ( Claussen *et al.*, 2013; Davies & Glasser, 2014; Evans, 2015; Kietzmann *et al.*, 2011). Our efforts so far have focused on three platforms: Twitter, Facebook, and ScoopIt. As described by Van Noorden (2014), Twitter and Facebook are perceived very differently, with far more of the scientists they surveyed indicating that professionally they prefer Twitter to Facebook. Our data indicate that the geographic distribution of people “following” or “liking” the Teaching Tools in Plant Biology feed differs between the two platforms ( Figure 5). Notably, more than two thirds of Twitter followers are from the UK, the US, Canada, and Australia, a fact that emphasizes the importance of language for this type of interaction. By contrast, seven out of eight of the largest groups of followers of the Facebook feed are from countries whose primary languages are not English.

The geographically broad distribution of followers of both the Teaching Tools articles and the social media feeds provides an interesting challenge for the content curators and creators, which is to be vigilant about the tone and implications of the materials shared. Many companies and organizations have developed social media guidelines for their staff to follow ( http://socialmediagovernance.com/policies/), and these are particularly relevant when considering diverse audiences. It is possible for a tweet that is meant to be ironic or humorous to be wildly misinterpreted, and there are plenty of well-publicized social media mistakes to serve as reminders of the importance of the rule “think before you tweet”. Trust is one of the most important factors for the success of online learning, so providing a positive online presence has been one of our goals ( Wang, 2014).

A successful online resource has to reflect the values and concerns of a broadly multicultural community ( Liu *et al.*, 2010). To further support the global community of plant scientists, we endeavour to use examples and case studies highlighting agricultural challenges from geographically diverse regions. Recent Teaching Tools describe the challenges of seawater incursion in Bangladesh, groundwater limitations in northern China, and phosphate-deprived soils of Chile and South Western Australia. Contributors to our social media communities are excellent sources of information about regional concerns and perspectives.

Finally, it is clear that our social media platforms are helping to connect the core materials to our audience. As an example, on December 30 ^th^, 2014, we posted a new Teaching Tool. Forty-eight hours after posting a notice of this new tool on Facebook, the notice had been seen by >3300 people, shared 30 times, and been “liked” 56 times, with more than 500 pageviews of the new article.

## Moving forward – Supporting interaction and engagement

Through social media, we are creating an environment in which professors, postdocs, and students can share and discuss ideas about current research in plant biology. The platforms we are using now arrange the information chronologically, but later this year we are launching a custom-built platform that will allow the discussions to be archived by theme and topic as well. We are designing the new platform to encourage direct peer-to-peer sharing of teaching tips and strategies. For example, many users of Teaching Tools ask us to suggest videos or animations to accompany the topics, and we will include a simple user interface to support uploading links to such materials. We envision that when an instructor or student is ready to learn “what’s new in photosynthesis”, he or she can find not only the relevant Teaching Tool, but also links to more recent articles, animations, lesson plans, and teaching ideas contributed from the community. A similar model for introductory biology topics was recently unveiled ( CourseSource.org). Another benefit of migrating our discussions and curated content to a custom site is that large social-networking sites such as Facebook and Twitter are blocked for users in China, yet a sizable proportion of Teaching Tools in Plant Biology users are in China and China has experienced rapid growth in the plant science research community in the past two decades ( Chen *et al.*, 2006); this dedicated site should enable educators and learners in China to participate in the global plant science learning community. 

## Does one size fit all? Should it?

Our own experiences and several other studies suggest that there are many different ways that teachers and learners can use online educational materials, both in and out of the formal classroom environment. Social and cultural issues, centralized control of curriculum and exams, access to textbooks, and fluency in English all affect the learning experience. Support has been strong for the approach we have taken, which is to make available a diversity of materials suitable for teachers and learners to use as they will. Our strategy has been to create and curate high-quality, authoritative content that highlights and interprets the cutting-edge of plant science, and we are increasingly providing an opportunity for educators and learners to share in the development of these resources. We are confident that through Teaching Tools in Plant Biology we are making a significant contribution to developing the next generation of educational tools with global outreach.

## References

[ref-1] Adams CT Hemingway CA: What does online mentorship of secondary science students look like? *BioScience.* 2014;64(11):1042–1051. 10.1093/biosci/biu147

[ref-2] American Association for the Advancement of Science: Vision and Change in Undergraduate Biology Education: A Call to Action.2011 Reference Source

[ref-3] Bik HM Goldstein MC: An introduction to social media for scientists. *PLoS Biol.* 2013;11(4):e1001535. 10.1371/journal.pbio.1001535 23630451 PMC3635859

[ref-4] Brown RE: The process of community-building in distance learning classes. *J Asynchron Learning Networks.* 2001;5:18–35. Reference Source

[ref-5] Chen H Karplus VJ Ma H: Plant biology research comes of age in China. *Plant Cell.* 2006;18(11):2855–2864. 10.1105/tpc.106.045393 17170389 PMC1693935

[ref-6] Claussen JE Cooney PB Defilippi JM: Science communication in a digital age: Social media and the American Fisheries Society. *Fisheries.* 2013;38(8):359–362. 10.1080/03632415.2013.816289

[ref-7] Davies BJ Glasser NF: Analysis of www.AntarcticGlaciers.org as a tool for online science communication. *J Glaciol.* 2014;60(220):399–406. 10.3189/2014JoG13J194

[ref-8] Estavillo GM Mathesius U Djordjevic M: The Plant Detective’s Manual: A research-led approach for teaching plant science. Australian National University; Canberra.2014 Reference Source

[ref-9] Evans P: Open online spaces of professional learning: Context, personalisation and facilitation. *TechTrends.* 2015;59(1):31–36. 10.1007/s11528-014-0817-7

[ref-10] Freeman S Eddy SL McDonough M: Active learning increases student performance in science, engineering, and mathematics. *Proc Natl Acad Sci U S A.* 2014;111(23):8410–8415. 10.1073/pnas.1319030111 24821756 PMC4060654

[ref-11] Hemingway C Adams C Stuhlsatz M: Digital collaborative learning: identifying what students value [v1; ref status: indexed, http://f1000r.es/55h]. *F1000Res.* 2015;4:74. 10.12688/f1000research.6223.1 26097690 PMC4457119

[ref-12] Internet World Stats: Internet Users by Country.2014 Reference Source

[ref-13] Jenkins DM: Plants – an ideal living material for teaching science. *School Science Review* In press,2015 Reference Source

[ref-14] Kietzmann JH Hermkens K McCarthy IP: Social media? Get serious! Understanding the functional building blocks of social media. *Business Horizons.* 2011;54(3):241–251. 10.1016/j.bushor.2011.01.005

[ref-15] Lee D: Web-based instruction in China: Cultural and pedagogical implications and challenges. *Educ Technol Res Devel.* 2004;52(1):101–105. 10.1007/BF02504779

[ref-16] Levesley A Jopson J Knight C: The Gatsby Plant Science Summer School: inspiring the next generation of plant science researchers. *Plant Cell.* 2012;24(4):1306–1315. 10.1105/tpc.111.094326 22534129 PMC3398476

[ref-17] Levesley A Paxton S Collins R: Engaging students with plant science: the Plant Science TREE. *New Phytol.* 2014;203(4):1041–1048. 10.1111/nph.12905 24942801

[ref-18] Liu X Liu S Lee SH: Cultural differences in online learning: International student perceptions. *Educ Technol Soc.* 2010;13(3):177–188. Reference Source

[ref-19] Mosaic Communications Press Release.1994 Reference Source

[ref-20] Osterrieder A: The value and use of social media as communication tool in the plant sciences. *Plant Methods.* 2013;9(1):26. 10.1186/1746-4811-9-26 23845168 PMC3716900

[ref-21] Quinn H Schweingruber H Keller T, eds: A framework for K-12 science education: Practices, crosscutting concepts, and core ideas. National Academies Press2012 Reference Source

[ref-22] Ray DK Mueller ND West PC: Yield trends are insufficient to double global crop production by 2050. *PLoS One.* 2013;8(6):e66428. 10.1371/journal.pone.0066428 23840465 PMC3686737

[ref-23] Rovai A: Building sense of community at a distance. *Int Rev Res Open Distrib Learning.* 2002;3(1):1–16. Reference Source

[ref-24] Tanner KD: Talking to learn: Why biology students should be talking in classrooms and how to make it happen. *CBE Life Sci Edu.* 2009;8(2):89–94. 10.1187/cbe.09-03-0021 19487494 PMC2689152

[ref-25] Thompson L Ku HY: Chinese graduate students' experiences and attitudes toward online learning. *Educ Media Int.* 2005;42(1):33–47. 10.1080/09523980500116878

[ref-26] Van Noorden R: Online collaboration: Scientists and the social network. *Nature.* 2014;512(7513):126–129. 10.1038/512126a 25119221

[ref-27] Wang YD: Building student trust in online learning environments. *Distance Education.* 2014;35(3):345–359. 10.1080/01587919.2015.955267

[ref-28] Zhang J: A cultural look at information and communication technologies in Eastern education. *Educ Technol Res Devel.* 2007;55(3):301–314. 10.1007/s11423-007-9040-y

[ref-29] Zhang J: Technology-supported learning innovation in cultural contexts. *Educ Technol Res Devel.* 2010;58(2):229–243. 10.1007/s11423-009-9137-6

