# Supplementary material for: Digital teaching tools and global learning communities
Source: F1000Res. 2015 Jul 20;4:59. Originally published 2015 Mar 5. [Version 2] doi: 10.12688/f1000research.6150.2 (PMC4406190; doi:10.12688/f1000research.6150.2)
Supplement: Peer review history

# Peer review: Referee response for version 1

**DOI:** 10.5256/f1000research.6591.r8187

**Published:** 2015-04-08

**Authors:** Mirza Hasanuzzaman

**Affiliations:** 1 Department of Agronomy, Faculty of Agriculture, Sher-e-Bangla Agricultural University, Dhaka, Bangladesh

This is a nice piece of work which examines how the produced materials are being used by different communities. The authors presented the distribution of the followers of Teaching Tools in Plant Biology on social networks. But in some countries some networks (e.g. Facebook in China) may not be available. How have the authors considered this?

I have read this submission. I believe that I have an appropriate level of expertise to confirm that it is of an acceptable scientific standard.

# Peer review: Referee response for version 1

**DOI:** 10.5256/f1000research.6591.r7863

**Published:** 2015-04-07

**Authors:** Claire Hemingway

**Affiliations:** 1 Division of Graduate Education, National Science Foundation, Arlington, VA, USA

The authors present findings on users of a suite of peer-reviewed digital educational materials available to subscribers of *The Plant Cell* and describe the efforts to develop this global community. This review of the Teaching Tools in Plant Biology’s user community is timely and important for several reasons.

The importance of this article lies in part on the very fact of the Teaching Tools in Plant Biology’s existence.  Launching the delivery of university-level digital teaching and learning materials with this high impact journal was a bold venture. Scientific societies that publish journals are faced with altered publishing economies as the landscape shifts from subscription based to open access models. At the same time, most scientific societies are also seeking to meet their education mission in ways that have significant impact and potential to be sustained. The approach to publish education articles with a society’s flagship journal remains relatively rare today. Thus, *The Plant Cell* and the American Society of Plant Biologists deserve recognition for initiating this practice in 2009.

As an opinion piece, the authors present a compelling argument that the Teaching Tools in Plant Biology are needed resources. The website analytics document the global reach of the teaching and learning materials.  But the major contribution of this article is the user survey results, which shows the richer picture of who uses the resources, in what ways, and for what reasons. While the authors report that about half of the 300 survey respondents were instructors in a course and half were students enrolled in a course or free-choice learners, more information about the demographics of the respondents would have been useful. The tantalizing finding about the influence of institutional, political, and cultural practices as well as the instructor preferences on material use deserves additional attention as the project moves towards its next phase of harnessing social media to develop a global learning community.

My primary reservation with this opinion piece is the lack of discussion of digital learning resources, and the trend over the years to shift away from digital repositories of resources to communities of practice engaged around the resources. The article frames the introduction in the general context of the radical changes the internet has brought to the ways educational tools are accessed and used. Then in discussing the future directions, the authors briefly mention the open-access, peer-reviewed journal of evidence-based curricula available at CourseSource. Although I would have liked the authors to present a view of how their materials fit within the constellation of digital learning resources available today, this opinion piece and the Teaching Tools in Plant Biology are a valuable contribution.

I have read this submission. I believe that I have an appropriate level of expertise to confirm that it is of an acceptable scientific standard.
